# Problem-Solving Deficits in Iranian People with Borderline Personality Disorder

**Published:** 2014

**Authors:** Ashraf Akbari Dehaghi, Hossein Kaviani, Shima Tamanaeefar

**Affiliations:** 1 Clinical Psychology, Department of Clinical Psychology, Tehran Psychiatric Institute, Tehran University of Medical Sciences, Tehran, Iran.; 2 Clinical Psychologist, Senior Lecturer in Clinical Psychology, Department of Psychology, University of Bedfordshire, Bedfordshire, United Kingdom.; 3 PhD Student, Department of Clinical Psychology, Tehran Psychiatric Institute, Tehran University of Medical Sciences, Tehran, Iran

**Keywords:** Borderline Personality Disorder, Female, Problem Solving, Suicide

## Abstract

**Objective:**

Interventions for people suffering from borderline personality disorder (BPD), such as dialectical behavior therapy, often include a problem-solving component. However, there is an absence of published studies examining the problem-solving abilities of this client group in Iran. The study compared inpatients and outpatients with BPD and a control group on problem-solving capabilities in an Iranian sample. It was hypothesized that patients with BPD would have more deficiencies in this area.

**Methods:**

Fifteen patients with BPD were compared to 15 healthy participants. Means-ends problem-solving task (MEPS) was used to measure problem-solving skills in both groups.

**Results:**

BPD group reported less effective strategies in solving problems as opposed to the healthy group. Compared to the control group, participants with BPD provided empirical support for the use of problem-solving interventions with people suffering from BPD.

**Conclusions:**

The findings supported the idea that a problem-solving intervention can be efficiently applied either as a stand-alone therapy or in conjunction with other available psychotherapies to treat people with BPD.

## Introduction

Borderline personality disorder (BPD) is characterized by a pervasive pattern of instability in interpersonal relationships, deregulated affectivity, negative self-image, and marked behavioral impulsivity. Among these characteristics, affective dysregulation is postulated to be a central mechanism of the disorder (1). One of the basic assumptions of dialectical behavior therapy (DBT) is that people with BPD have deficits in their problem-solving abilities (2). Linehan postulated that people with BPD either do not have the necessary skills to adequately solve problems or are unable to use these skills due to their heightened emotional state. That is why problem-solving skills training is therefore one of the major components of DBT, along with mindfulness training and strategies aimed at enhancing affective regulation (2). Effective social problem-solving can increase situational coping and behavioral competence, which in turn, may prevent or reduce emotional distress (3). A negative problem-solving orientation includes cognitions and emotions that are deemed to inhibit adaptive problem-solving (4).

To explain this phenomenon more precisely, we may need to mention the link between problem-solving and autobiographical memory retrieval. The research shows that over-general memory retrieval is associated with downstream impairments in problem-solving, which are known to increase suicide risk (5, 6). One of the contributing factors in problem solving deficits among suicidal patients is a difficulty in retrieval of autobiographical memory (7-9). In terms of clinical implications for everyday life, over-general tendency in autobiographical memory might give rise to poor problem-solving and difficulties in imagining the future (10, 11). 

There seems to be an overlap between symptoms observed in people with BPD and parasuicides; which encompasses self-harming behaviors, emotional instability, problems in anger management, interpersonal problems and low self-esteem (1). Thus, Linehan (1) suggested that it might be feasible to generalize the findings from one group of patients to another. In line with this, Kehrer and Kehrer (12) identified inappropriate problem-solving strategies to be predictive of parasuicidal behavior in women who met the criteria for BPD. McMurran et al. (13) have proposed a model in which the concept of social problem solving is central to adaptive functioning. In fact, in order to solve a problem rationally, we need first to define the problem, then set the goals, generate alternative options, analyze options, make a decision, and finally implement the solution decided up on. These are core skills to both the social problem solving model of personality disorder and to social problem solving therapy.

McMurran et al. (13) claimed that innate traits are the developmental starting point for behavioral patterns. Specific personality traits restrict and bias information processing, intervene with learning social problem-solving skills, and probably lead to dysfunctions in everyday life. Interpersonal dysfunction causes distress, which may give rise to anxiety, depression and anger. Stress further impairs problem-solving abilities and may also prepare the ground for problematic and maladaptive behaviors to ease stress, such as substance use, which per se, impairs social problem-solving abilities and furthermore creates additional interpersonal problems. This vicious circle leads to a negative approach to life’s problems and the growth of maladaptive self-schemas that have a further harmful effect on information processing and social problem solving (14). Furthermore, problem-solving orientation may predict the probable effect of treatment, especially with regards to suicidal actions. Patients who are positively problem-solving oriented, are more likely to benefit from treatment than patients with low positive problem orientation (4). There is plenty of research conducted to examine problem-solving abilities of psychiatric patients who attempted suicide (2, 15-18). Deficits in this field have also been found in other patient populations, including people suffering from depression and anxiety disorders (6, 8, 19). These studies mostly employed either the Social Problem-Solving Inventory-Revised (SPSI-R) (20) to assess social problem solving or Means-End Problem Solving (MEPS) (21). To date, the problem-solving abilities of Iranian people with BPD have not been studied. In this study, we used MEPS which has been used by other groups (7-9, 22) and has been translated in Persian and validated in Iranian participants, and even used in studies in depressed patients with and without suicide ideation and also in parasuicide patients (6, 18). As the aim of this study was to investigate the problem solving abilities of people who met the diagnostic criteria for BPD, it was hypothesized that participants with BPD: 1. produce less effective solutions; 2. provide fewer number of means; 3. provide more irrelevant means; and 4. take longer to respond to the task and all have been compared with the performance of healthy control group.

## Materials and Methods

***Participants***

In a cross-sectional study, 15 female patients with BPD (mean age = 23.13) standard deviation (±SD) = 4.03 years and 15 healthy female participants (mean age = 22.66; ±SD = 4.20 years), matched for age (±5 years), education and marital status were tested. Both groups were recruited from an accessible as well as convenient sample. Demographic variables are displayed in table 1. Using the semi-structured clinical interview of Diagnostic and Statistical Manual of Mental Disorders (DSM-IV), Axis II (SCID-II) for personality disorders (23), a psychiatrist evaluated patients meeting DSM-IV criteria (24) for BPD. The patients with comorbid disorders, including major depressive disorder, schizophrenia, obsession-compulsion and bipolar disorder were excluded. They were also screened against the conspicuous brain lesions, electroconvulsive therapy (ECT) in the last six months and the criteria of substance abuse. On the basis of the interview, cases with any sign of altered consciousness secondary to medication overdose were also excluded from the experiment (one case). More than a half of the patients had either committed suicide or had threatened to attempt suicide at least once in the past. Inpatient samples were selected from Hazrat-e-Rasool Hospital which is a university hospital that has a psychiatric ward. Suicidal and other psychiatric cases are referred to this ward for appropriate treatments. Outpatient BPD samples were selected by psychologists and psychiatrists from the referral clients (clinicians became aware by advertisement). The clinical sample included 5 outpatients and 10 inpatients. Seven of ten inpatients had suicidal tendencies. All outpatients were dweller in Tehran (capital of Iran) but inpatients were from Tehran and other provinces of Iran. Healthy participants were mostly hospital staff or general library clients in Tehran city that were screened using inclusion and exclusion criteria. 

***Materials and measures***

*Demographic characteristics questionnaire*: this questionnaire was designed to detect basic demographical data including age, education level, marital status, occupation status, and background of any referral to a psychologist or psychiatrist.

*The Structured Clinical Interview for DSM-IV Axis II (SCID-II)*: The SCID-II (25) is a semi-structured diagnostic interview that covers all 13 personality disorders described in DSM-IV. Each participant completed the SCID-II personality questionnaire to identify potential personality disorders and then the diagnosis was confirmed or disconfirmed using the SCID-II interview. The SCID-II is widely used in clinical research and has been reported to have adequate psychometric properties (26). Bakhtiari research (27) showed the content validity and test-retest reliability (r = 0.86) of the SCID in an Iranian population.

*Means-Ends Problem Solving Task (MEPS)*: Looking at the problem-solving task presented by Platt et al. (21), a similar and culturally modified task including five situations was developed in a previous study (18). This task was used in the present study to measure different aspects of problem-solving in the target population. The participant was presented with each situation that consisted of a story with a stated need and a desired end. The respondent was asked to complete the middle part of the story so that the protagonist would achieve the desired outcome.

In the present study, a number of suitable components for the aims of the study were scored: namely, latency to response onset (first word of response), number of means (Including relevant means and irrelevant means), relevancy ratio (relevant means/number of means), and means effectiveness. Effectiveness rating was determined using the criteria of Evans et al. (7). To quantify effectiveness, the two independent raters (one blind to the status of the participants) used a 3-point scale (0 = not at all effective; 1 = effective; 2 = very effective). This procedure yielded good inter-rater agreement, i.e. no substantive disagreement. Mean rater scores were used in the analysis. The validity and reliability of the Persian version of MEPS has been piloted by Kaviani et al. (18).

**Table 1:** Demographical variables of patients with borderline personality disorder (n = 15) and healthy group (n = 15)

**Variable**	**Patients with borderline personality disorder**			**Healthy subjects**	
	**Variable levels**	**Frequency**	**Percentage**	**Frequency**	**Percentage**
**Age**	18-20	2	13.3	6	40
	21-25	12	80	7	46.7
	26-30	-	-	1	06.7
	31-35	1	6.8	1	06.7
**Occupation**	Employed	5	33.3	3	20
	Unemployed	9	60	10	66.7
	Student	1	6.7	2	13.3
**Marital status**	Single	13	86.7	14	93.3
	Married	2	13.3	1	06.7
**Education**	High school graduated	2	13.3	1	06.7
	Diploma	9	60	10	66.7
	Bachelor	3	20	3	20
	Master degree	1	6.7	1	06.7

***Procedure***

All the potential participants were given information about the study and volunteers gave their written consent. Only one patient with BPD refused to take part in the experiment. Prior to assessments, volunteers were screened in relation to the inclusion and exclusion criteria. Participants were tested individually. Testing was done in two sessions, with the time interval of 1 to 2 days. Session 1 consisted of filling in the informed consent, and administering SCID; session 2 consisted of conducting the MEPS. The testing time in session 2 was between 20 to 35 min. Author “a” executed test as the first rater, the author “c” was the second rater who was blind to the rating of the experimenter's. Correlations coefficients between the first and second raters were significant (relevancy ratio r = 0.80, effective strategies r = 0.62, and number of means r = 0.70).

## Results

In general, the interviews using SCID-II showed that the participants with BPD have deficits in all the criteria as the following: frantic efforts to avoid abandonment (66%), a pattern of disturbed interpersonal relationship (92%), identity disturbance (83%), impulsivity (50%), recurrent suicide or suicide threats (75%), affective instability (66%), feeling of emptiness (100%), intense anger (66%), and paranoid ideation (58%).

A series of t-tests were performed to compare group performance on different variables measured in problem-solving tasks. Table 2 shows statistics of problem-solving measures (latency, number of means, and number of irrelevant means, relevancy ratio, and effectiveness) in both BPD and control groups. 1. It was hypothesized that participants with BPD produce less effective solutions; the findings that compared to the control group, the BPD group provided less effective strategies (t = 4.47, p < 0.001). 2. It was also hypothesized that people with BPD provide fewer number of problem-solving means; the findings show fewer means in this sample (t = 3.43, p < 0.001). 3. Another hypothesis was that the patients with BPD provide more irrelevant means. Findings of this study support this hypothesis and show a significant differentiation between the two groups and their data are depicted in table 2: (t = 3.91, p < 0.001). 4. The next hypothesis indicated that the participants with BPD take longer to respond to the task, compared to the performance of the healthy control group. Table 2 shows the BPD group took longer to respond to the task than the matched healthy control participants (t = 2.29, p < 0.05). Moreover, table 2 shows a lower relevancy ratio in the people with BPD than the healthy control group (t = 4.10, p < 0.001). 

Table 3 shows correlations between different variables. There was a positive correlation between the number of means and relevancy ratio (r = 0.85, p < 0.01); also a significant positive correlation between the number of means and effectiveness (r = 0.93, p < 0.01). In addition, as shown in table 3, there were significant negative correlations between number of irrelevant means with number of means (r = -0.60, p < 0.01); with relevancy ratio (r = -0.73, p < 0.01) and with effectiveness (r = -0.63, p < 0.01). Furthermore, there was a significant correlation between the relevancy ratio and effectiveness (r = 0.89, p < 0.01).

**Table 2:** Comparison of the two studied groups (borderline personality disorder (BPD) vs. healthy subjects) regarding problem-solving measures

**Measure **	**BPD** † ** (n = 15)** **Means (±SD)**	**Control (n = 15)** **Means (±SD)**	**t**
**Number of means**	4.73 (±1.94)	8.00(±3.21)	3.43**
**Number of irrelevant means**	2.00 (±1.81)	0.13 (±0.35)	3.91**
**Relevancy ratio**	2.36 (±1.00)	3.88 (±1.01)	4.10**
**Effectiveness**	3.06 (±1.53)	7.13 (±2.16)	4.47**
**Latency**	45.26 (±34.67)	20.33 (±23.87)	2.29**

* p < 0.05;

** p < 0.001;

†: borderline personality disorder

**Table 3:** Correlation between components of Means-Ends Problem Solving Task (MEPS)

	**Number of irrelevant means**	**Relevancy ratio**	**Effectiveness**	**Latency**
**Number of means**	-0.60**	-0.85**	-0.93**	-0.19
**Number of irrelevant means**		-0.73**	-0.63**	-0.11
**Relevancy ratio**			-0.89**	-0.22
**Effectiveness**				-0.27
**Latency**				

** p < 0.01

## Discussion

As predicted, this study found that people with BPD experience deficits in problem-solving tasks. They provide solutions that contain fewer relevant means, which are less effective than those provided by the control group. The present findings have potential to empirically support one of the underlying assumptions in dialectical behavior therapy (DBT). Linehan (1) asserts that people with BPD have deficits in their problem-solving. In this respect, it might be appropriate to offer a problem-solving intervention to this group, either as a stand-alone therapy or in conjunction with other available psychotherapies (2). The similarities in problem-solving ability between the BPD group and the clinical control group suggest that it may be suitable for employing a problem-solving intervention similar to those developed for other client groups (28) provided that their specific problem-solving deficits are taken in to account. 

Two groups were similar in terms of age. The issue of possible rater’s bias was controlled by the second ratings; in other words, the high inter-rater correlation coefficient rules out the experimenter bias. 

This study shows that people with BPD provided fewer relevant means, less effective and relevant solutions than those in the control group. This is consistent with what Bray et al. (2) found as he extended an understanding of such cognitive aspects in a different cultural setting. These findings are in line with the results of other studies carried out in the same cultural setting, for example Kaviani et al. study (18), all having been done on suicidal depression patients. Deficits assessed by MEPS have also been found in other clinical populations, including people suffering from depression and anxiety disorders (6, 8, 19) and patients with schizophrenia (29). People with parasuicidal behavior or with severe suicide attempts generally generate fewer potentially effective steps to solve problems, their solutions are less effective and they are less likely to implement the solutions they generate (7, 17). They have lower expectations regarding their problem-solving abilities (15, 16). The present study has shown that patients with BPD reported fewer effective means in problem-solving than the controlled group. Reviewing existing literature, one might find out that training problem solving skills and specific memory retrieval skill proved to result in an improvement in over-general retrieval. An association was displayed between unspecific memory and poor problem-solving in the BPD group (30). Decreased specificity of autobiographical memory was further related to poor social problem-solving capacity in the BPD group. 

The results of this study have implications for clinical practice. These results suggest potential ways in which social problem solving therapy might be adapted in the clinical arena so as to target specific dysfunctions as social personality difficulties. When interpreting the results, there seem to be some limitations in this research including limited number of BPD patients who do not suffer any other disorders according to DSM-IV in axis I. Further research is set to be carried out on male patients with this disorder in order for the findings to be generalized. The results of this study can be regarded as a step to identify cognitive deficits in people suffering from BPD. In addition, the findings empirically support the use of problem-solving interventions to help people suffering from BPD. Goddard et al. (8) proposed that problems with the encoding, storage and retrieval of specific memories result in a limited knowledge base to draw upon to solve current social dilemmas. This association has important implications for the development of problem-solving interventions, as it implies that teaching people ways to improve the encoding and retrieval of their autobiographical memories may increase the effectiveness of their problem-solving attempts.

## Notes

**Declaration of interest:** None.
